# Random kernel k-nearest neighbors regression

**DOI:** 10.3389/fdata.2024.1402384

**Published:** 2024-07-01

**Authors:** Patchanok Srisuradetchai, Korn Suksrikran

**Affiliations:** Department of Mathematics and Statistics, Thammasat University, Pathum Thani, Thailand

**Keywords:** bootstrapping, feature selection, k-nearest neighbors regression, kernel k-nearest neighbors, state-of-the-art (SOTA)

## Abstract

The k-nearest neighbors (KNN) regression method, known for its nonparametric nature, is highly valued for its simplicity and its effectiveness in handling complex structured data, particularly in big data contexts. However, this method is susceptible to overfitting and fit discontinuity, which present significant challenges. This paper introduces the random kernel k-nearest neighbors (RK-KNN) regression as a novel approach that is well-suited for big data applications. It integrates kernel smoothing with bootstrap sampling to enhance prediction accuracy and the robustness of the model. This method aggregates multiple predictions using random sampling from the training dataset and selects subsets of input variables for kernel KNN (K-KNN). A comprehensive evaluation of RK-KNN on 15 diverse datasets, employing various kernel functions including Gaussian and Epanechnikov, demonstrates its superior performance. When compared to standard KNN and the random KNN (R-KNN) models, it significantly reduces the root mean square error (RMSE) and mean absolute error, as well as improving R-squared values. The RK-KNN variant that employs a specific kernel function yielding the lowest RMSE will be benchmarked against state-of-the-art methods, including support vector regression, artificial neural networks, and random forests.

## 1 Introduction

The recent increase in machine learning research has highlighted the significance of ensemble techniques and regression models, which have demonstrated enhanced predictive capabilities. This trend is observable across a wide range of domains and use cases, as evidenced by the current research landscape. Li et al. (2023) conducted a comprehensive study in the field of agriculture, analyzing meteorological patterns and soybean yield statistics from various counties and weather stations within China's primary soybean cultivation regions. They utilized a stacking ensemble framework to construct a predictive model for soybean yield estimation, employing algorithms such as k-nearest neighbor (KNN), random forest (RF), and support vector regression (SVR). Jiang et al. (2023) developed a stacking ensemble model that integrates RF, KNN regression, gradient boosting regression (GBR), and a meta-learner, specifically linear regression (LR), to predict greenhouse gas emissions from irrigated rice farms. Bian and Huang (2024) developed a novel fuzzy modeling approach using an enhanced evidence theory integrated with KNN for dynamic and accurate air pollution estimation.

In the energy sector, El-Kenawy et al. (2021) introduced an improved ensemble model for predicting solar radiation levels. This model operates in two stages: data preparation and ensemble training. It is enhanced through KNN regression, and its effectiveness is evaluated using a dataset from Kaggle. Compared to existing benchmarks, the unique advantages of this model are evident. In a related study, Chung et al. (2019) explored various machine learning techniques to predict charging patterns, analyzing factors such as duration and energy consumption from historical data. They developed the Ensemble Predicting Algorithm (EPA) by integrating diverse techniques to enhance predictive accuracy. Sharma and Lakshmi (2023) proposed a model that initially segments the values of the target variable into multiple categories. Then, a unified KNN model, which merges both weighted attribute KNN and distance-weighted KNN, is applied. The weighting for each attribute is determined through information gain. This model is employed to predict the target variable's value for each test instance. Their primary aim was to use various KNN-focused models to increase the accuracy of air pollutant level predictions. Cheng et al. (2014) introduced a novel KNN methodology based on sparse learning, designed to address the limitations of previous KNN approaches, such as using a fixed *k* value for all test instances and overlooking sample correlations. This strategy adjusts test samples and uses training samples to identify the optimal *k* value for each instance. Subsequently, the refined KNN method, with the optimized *k* value, is applied to various tasks, including categorization, regression, and imputation of missing values.

Song and Choi (2023) introduced innovative integrated models within the finance industry, aimed at forecasting both short-term and long-term closing prices of major stock market indices: DAX, DOW, and S&P500. They proposed an enhancement involving the calculation of the mean of the highest and lowest prices of these indices to improve accuracy. In a separate domain, Dimopoulos et al. (2018) conducted a comparative study on the effectiveness of machine learning vs. traditional risk ratings in estimating the risk of cardiovascular disease.

KNN regressions have also been discovered for environmental research. Jafar et al. (2023) conducted a study to compare the effectiveness of multiple linear regression with 19 different machine learning techniques. These algorithms included regression, decision trees, and boosting mechanisms. The analyzed models included LR, least angle regression (LAR), Bayesian ridge chain (BR), ridge regression (Ridge), KNN, extra tree regression, and the notably robust XGBoost. In a related effort, Srisuradetchai and Panichkitkosolkul (2022) employed an ensemble machine learning approach that incorporated KNN, MLR, RF, SVR, and other algorithms to predict PM2.5 levels in Bangkok. This ensemble learning method was further applied by Srisuradetchai et al. (2023) to forecast daily new confirmations of COVID-19 cases.

KNN regression has been enhanced through its combination with other algorithms. Ghavami et al. (2023) introduced an innovative ensemble prediction technique named COA-KNN, which integrates the Coyote optimization algorithm (COA) with KNN to enhance the accuracy of fatigue and rutting predictions in reclaimed asphalt pavement mixtures. When compared to established prediction models, including RF, GB, decision tree regression (DTR), and MLR, COA-KNN demonstrated superior performance across various metrics. Similarly, Song et al. (2018) developed a potent regression learning approach termed the distance-weighted KNN algorithm. This algorithm aims to elucidate the nonlinear relationships between input structural parameters and resultant motor performances.

In the expanding field of KNN classification, particularly in the context of big data, Bermejo and Cabestany (2000) pioneered an adaptive soft KNN classifier that estimates posterior class probabilities, showcasing improved handwritten character recognition. Meanwhile, Deng et al. (2016) optimized KNN classification for large datasets using a hybrid approach of k-means clustering and KNN classification. Ingram and Munzner (2015) proposed the Q-SNE algorithm, a dimensionality reduction technique tailored for document data, significantly enhancing the layout quality of large document collections. Similarly, Pramanik et al. (2021) reviewed the applications and challenges of big data classification, discussing the imperative of systematic data processing for knowledge discovery and decision-making. Saadatfar et al. (2020) addressed the computational challenges of applying KNN to big data by clustering data into smaller, manageable partitions. Abdalla and Amer (2022) introduced NCP-KNN, a variation that reduces search complexity and excels in high-dimensional classification, promising efficiency for large datasets. Finally, Ukey et al. (2023) delivered a comprehensive survey on exact KNN queries over high-dimensional data.

Kernel functions are employed in KNN, as demonstrated by Zheng and Cao (2008), who explored the use of kernel functions in KNN for Holter waveform classification. Enriquez et al. (2019) devised and examined a methodology for identifying faults in power transformers using a KNN classifier with a weighted classification distance. Rubio et al. (2009) introduced a parallel implementation of the sequential kernel-weighted KNN algorithm in Matlab, specifically designed for cluster platforms. Ali et al. (2020) developed a group model utilizing the KNN algorithm, employing samples and random features to generate predictions by pooling various models. Bay (1999) also explored a similar concept, aiming to enhance nearest neighbor classifiers through the utilization of a combination of multiple models, each emphasizing random features. However, these studies, including research conducted by García-Pedrajas and Ortiz-Boyer (2009), Steele (2009), and Li et al. (2014), primarily aimed to enhance classifiers by utilizing a random subset of input variables without considering the utilization of kernel functions. For the KNN time series model, Srisuradetchai (2023) proposed a new approach for interval forecasting that combines the KNN time series model with bootstrapping.

This study enhances random KNN regression by incorporating kernel methods. While traditional random KNN regression is effective with various data types, it may not detect intricate patterns that are crucial for accurate predictions. The method introduced here, named Random Kernel KNN regression (RK-KNN), employs random feature selection, bootstraps data samples, and applies kernel functions to weight distances. This paper evaluates RK-KNN across 15 datasets and compares its performance with state-of-the-art methods, including random forest, support vector regression, and artificial neural networks.

## 2 Theoretical background

### 2.1 Kernel functions

Kernel functions are used to weigh the contributions of each point based on its distance from the query point. While traditional KNN uses uniform weights, kernel functions allow these weights to vary, often improving performance. Here are some widely used kernels that can be applied in KNN regression (Schölkopf and Smola, 2001; Tsybakov, 2009; Beitollahi et al., 2022):

- Gaussian (Radial Basis Function) kernel:

Perhaps the most popular kernel, the Gaussian kernel, has a bell-shaped curve and can assign weights to points in the input space based on their distance from the query point, with this influence rapidly declining as the distance increases, as shown in Equation (1).


(1) $$\begin{array}{l} K\left(x,x^{\prime }\right)=\exp\left(-\frac{||x-x^{\prime }|{|}^{2}}{2\sigma^{2}}\;\right), \end{array}$$

where σ^2^ is the standard deviation (bandwidth).

- Epanechnikov kernel:

This kernel is parabolic and is often used because of its computational efficiency. It assigns more weight to nearby points than to points further away, but unlike the Gaussian kernel, it becomes zero beyond a certain distance, as defined in Equation (2).


(2) $$\begin{array}{l} K\left(x,x^{\prime }\right)=\frac{3}{4}\left(1-\frac{||x-x^{\prime }|{|}^{2}}{h^{2}}\right)for||x-x^{\prime }||<h, \end{array}$$

and *K*(*x, x*′) = 0 otherwise, where *h* is the bandwidth.

- Uniform kernel:

The uniform kernel gives equal weight to all points within a certain range of the query point and no weight to points outside this range. It is the simplest form of kernel and is equivalent to the traditional KNN method when used with a fixed radius, as expressed in Equation (3).


(3) $$\begin{array}{l} K\left(x,x^{\prime }\right)=\frac{1}{2h}\;for||x-x^{\prime }||<h,\; \end{array}$$

and *K*(*x, x*′) = 0 otherwise, where *h* is the bandwidth.

- Triangular kernel:

The triangular kernel assigns weights that decrease linearly with distance from the query point. It is zero beyond the kernel's bandwidth, as shown in Equation (4).


(4) $$\begin{array}{l} K\left(x,x^{\prime }\right)=1-\frac{\left||x-x^{\prime }|\right|}{h}\;for||x-x^{\prime }||<h, \end{array}$$

and *K*(*x, x*′) = 0 otherwise, where *h* is the bandwidth.

- Quartic (Biweight) kernel:

This kernel is similar to the Epanechnikov kernel but assigns weight with a smooth, bell-shaped curve, which reaches zero at the kernel's bandwidth, as defined in Equation (5).


(5) $$\begin{array}{l} K\left(x,x^{\prime }\right)=\frac{15}{16}{\left(1-{\left(\frac{\left||x-x^{\prime }|\right|}{h}\right)}^{2}\right)}^{2}for||x-x^{\prime }||<h,\; \end{array}$$

and *K*(*x, x*′) = 0 otherwise, where *h* is the bandwidth.

- Tricube kernel:

The tricube kernel is a higher-order kernel with compact support, meaning it assigns a weight of zero to any point outside a certain range of the query point. It is smoother and has heavier tails than the quartic kernel, according to Equation (6).


(6) $$\begin{array}{l} K\left(x,x^{\prime }\right)=\frac{70}{81}{\left(1-{\left(\frac{\left||x-x^{\prime }|\right|}{h}\right)}^{3}\right)}^{3}for||x-x^{\prime }||<h,\; \end{array}$$

and *K*(*x, x*′) = 0 otherwise, where *h* is the bandwidth.

**Figure 1:**
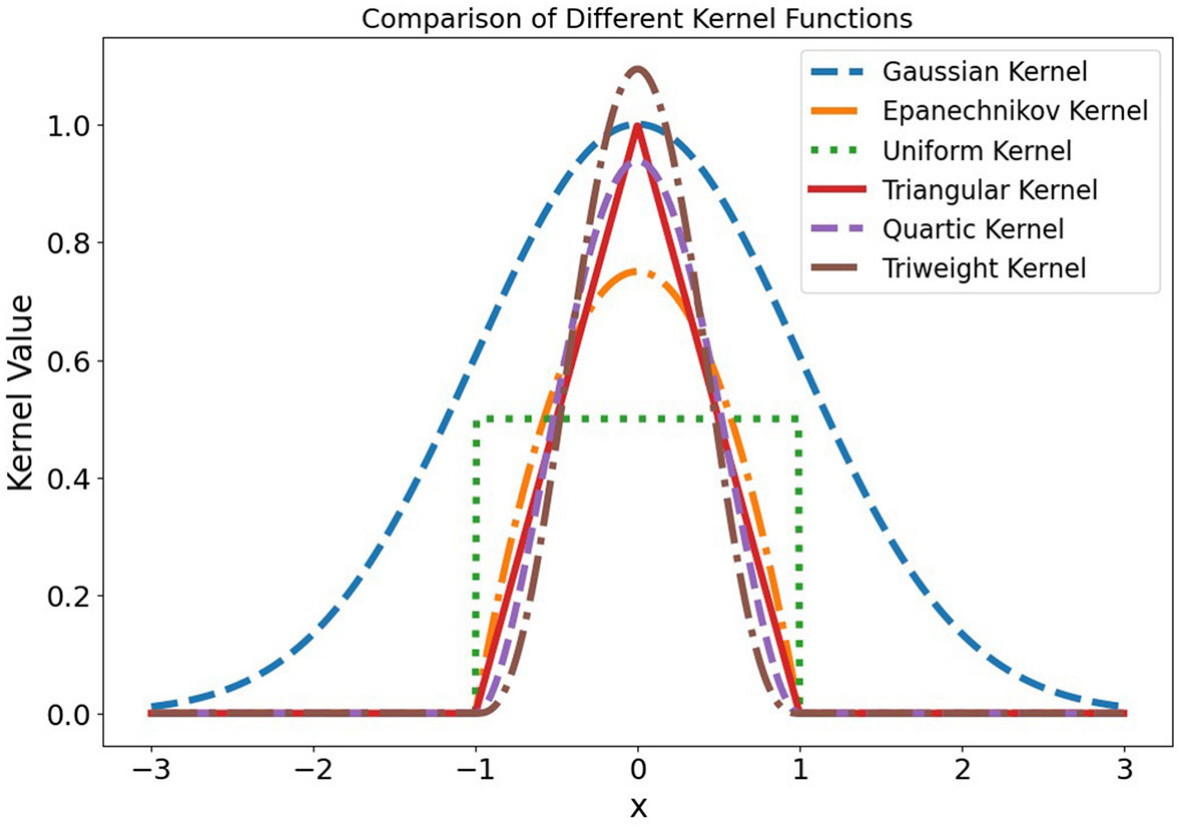
Comparison of different kernel functions, all centered at zero and using a bandwidth of one.

All kernel functions, as plotted in Figure 1, have a bandwidth of one. The Gaussian kernel is depicted as a smooth curve peaking at the center. The Epanechnikov kernel displays a parabolic shape that cuts off at the bandwidth's edge. The uniform kernel provides equal weight within a fixed bandwidth and falls to zero beyond it. The triangular kernel's weight decreases linearly with distance, ending at the bandwidth limit. The quartic kernel features a bell shape that smoothly tapers to zero, while the tricube kernel has a more pronounced peak with a faster decline.

### 2.2 K-nearest neighbor regression

KNN regression is a type of non-parametric method used for predicting the continuous outcome of a new data point based on the outcomes of its nearest neighbors in the feature space. It does not make any assumptions about the underlying data distribution and is particularly useful when dealing with complex data structures (Hastie et al., 2009). Given a dataset with *n* points, (*x*_1_, *y*_1_), (*x*_2_, *y*_2_), ..., (*x*_*n*_, *y*_*n*_), where each *x*_*i*_ represents a vector of features and each *y*_*i*_ represents the corresponding continuous outcome, KNN regression predicts the outcome ŷ for a new data point *x* based on the outcomes of its *k* nearest neighbors in the feature space. The mathematical formulation of KNN regression includes (Altman, 1992):

- Distance metric: the first step in KNN regression is to determine the “closeness” of data points in the feature space, which requires a distance metric. The most common choice is the Euclidean distance, though other metrics like Manhattan or Minkowski can also be used. The distance *d* between two data points *x* and *x*_*i*_ is calculated by using Equation (7) for Euclidean distance.
(7) $$\begin{array}{l} d\left(x,x_{i}\right)=\sqrt{{\left(x-x_{i}\right)}^{T}\left(x-x_{i}\right)} \end{array}$$
- Finding neighbors: for a given data point *x*, find the *k* points in the dataset that are closest to *x* based on the distance metric. These points are termed KNN.
- Prediction: The predicted outcome ŷ is calculated as the average of the outcomes of the k-nearest neighbors. Mathematically, this can be represented as:
(8) $$\begin{array}{l} ŷ=\frac{1}{k}\sum_{i\in N_{k}}y_{i}. \end{array}$$
  In Equation (8), *N*_*k*_ contains the indices of the *k* closest (in *l*_2_ distance) of *x*_1_, ..., *x*_*n*_ to *x*.

**Figure 2:**
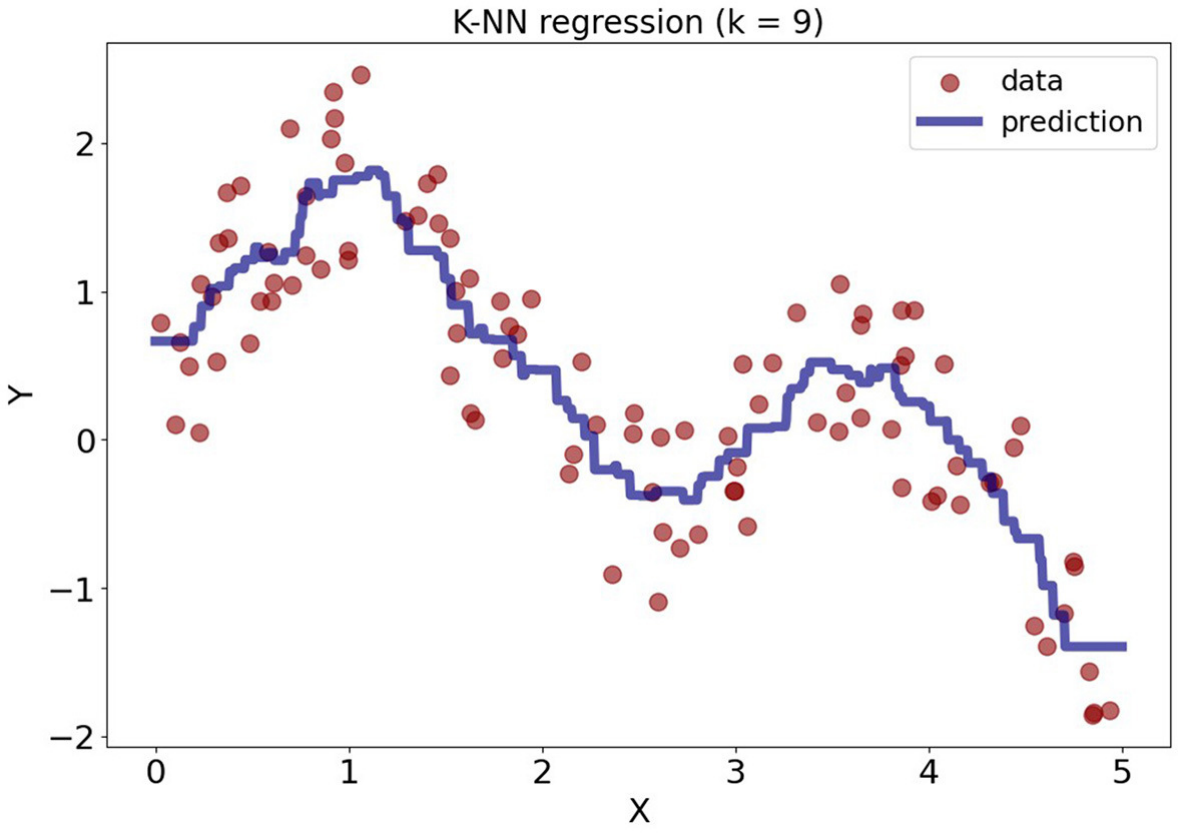
KNN regression model with *k* = 9 applied to synthetically generated data.

Figure 2 illustrates the example of the KNN regression with *k* = 10, where the dataset was synthetically generated from model *Y* = sin(*x*)+ sin(2*x*)+ε. It can be observed that KNN regression makes no assumptions about linearity and fits the data well. The predictions for new data points are based on the average outcomes of the 9 nearest points from the training data.

### 2.3 Kernel k-nearest neighbor regression

Kernel *k*-Nearest Neighbor (K-KNN) regression extends the conventional KNN regression algorithm, an instance-based learning method, by incorporating kernel functions. This integration allows the algorithm to weigh the contributions of each point's neighbors based on their distance, effectively smoothing out predictions and improving the model's ability to handle complex, non-linear relationships (Tan et al., 2020; Yao et al., 2021). Given a dataset with *n* points (*x*_1_, *y*_1_), (*x*_2_, *y*_2_), ..., (*x*_*n*_, *y*_*n*_), where each *x*_*i*_ represents a vector of features and each *y*_*i*_ represents the corresponding continuous outcome, the prediction ŷ for a new data point *x* is calculated not just by averaging the outcomes of its *k* but by taking a weighted average, where the weights are determined by a kernel function based on the distance between *x* and each *x*_*i*_.

The kernel function *K* in Equation (9) applied in this context is a symmetric function that satisfies certain mathematical conditions (like positivity and integrability) with the general form *K*:ℝ^*d*^ → ℝ, where *d* is the dimension of the input space. The kernel function *K* must satisfy


(9) $$\begin{array}{l} \int K\left(t\right)\;dt=1,\;\int t\;K\left(t\right)\;dt=0,\;0<\int t^{2}\;K\left(t\right)\;dt<\infty . \end{array}$$

The choice of kernel function can significantly influence the regression outcome, as different kernels impose different structures on the data (Hofmann et al., 2008). The prediction ŷ in K-KNN regression is then given by:


(10) $$\begin{array}{l} ŷ=\frac{\underset{i\in N_{k}}{\sum }K\left(x,x_{i}\right)\cdot y_{i}}{\underset{i\in N_{k}}{\sum }K\left(x,x_{i}\right)}. \end{array}$$

In Equation (10) *K*(*x, x*_*i*_) is the kernel function evaluating the similarity (or smoothness) between the target point *x* and each neighbor *x*_*i*_.

### 2.4 Cross-validation for optimal parameters

It is imperative to determine the optimal *k* for the neighbors and the best-suited bandwidth for the kernel function in the context of each bootstrap sample. This step ensures that the model is not just fitted to the training data but also generalizes well to unseen data.

Utilizing ν−fold cross-validation, the original training set is randomly partitioned into ν equal-sized subsamples. Of the ν subsamples, a single subsample is retained as the validation data for testing the model, and the remaining ν−1 subsamples are used as training data. The cross-validation process is then repeated ν times (the folds), with each of the ν subsamples used exactly once as the validation data. For each fold and each candidate combination of parameters (specific *k* and bandwidth), the model is trained, and the prediction error (e.g., RMSE) on the validation fold is computed. The average error across all ν folds is then calculated for each combination (Wong and Yang, 2017; Wong and Yeh, 2020).

## 3 Proposed method

Combining bootstrap sampling, choosing features at random, and using kernel methods in a KNN model is employed to make standard KNN better at prediction. Given a training dataset *LD*(*X*; *Y*), where *X* is a *p*-dimensional feature matrix with *n* observations and *Y* is the corresponding response variable, the objective is to predict the response ŷ for a new observation *x*_0_ in the test dataset. Note that in KNN regression, it is essential to preprocess all predictors to ensure they are unitless. The step for random kernel KNN (RK-KNN) regression is as follows:

1) *Bootstrap Sampling for KNN*

Bootstrap sampling is integral to ensemble methodologies, particularly bagging. It involves generating *B* unique datasets from the original training data, *D*, each termed *D*_*b*_ (where *b* = 1, 2, …, *B*) by sampling *n* observations with replacement. In mathematical notation,


(11) $$\begin{array}{l} D_{b}=\left\{\left(x_{1}^{{\;}^{*}},y_{1}^{{\;}^{*}}\right),\left(x_{2}^{{\;}^{*}},y_{2}^{{\;}^{*}}\right),\ldots ,\left(x_{n}^{{\;}^{*}},y_{n}^{{\;}^{*}}\right)\right\}. \end{array}$$

In this study, *B* is set to 1,000.

3) *Random Feature Selection*

Incorporating a feature randomness aspect akin to Random Forests, each bootstrap sample *D*_*b*_, as shown in Equation (11), undergoes a feature selection process where only a random subset of *d* features (where *d*<*p*) is considered for model training. During the training phase for each *D*_*b*_, the algorithm does not utilize the full feature set. Instead, it randomly selects a subset, contributing to model diversity within the ensemble (Breiman, 2001). In this study, *d* is set to *p*/2, *p*/5, and $\sqrt{p}$, and the best *d* is determined from one that yields the lowest RMSE or MAE or highest R^2^.

3) *Kernel Enhancement in KNN*

We add the Gaussian, Epanechnikov, uniform, triangular, quartic, and tricube kernels to a standard KNN in this paper. Within KNN, kernel functions can adjust neighbor contributions, giving more weight to nearer neighbors. Suppose *N*_*k*_(*x*_0_) denotes the set of *k* nearest neighbors to a query point *x*_0_, determined using a subset of *d* features. The kernel-weighted response estimate is given by:


(12) $$\begin{array}{l} ŷ=\frac{\underset{x_{i}\in N_{k}\left(x_{0}\right)}{\sum }K\left(x_{0},x_{i}\right)\cdot y_{i}}{\underset{x_{i}\in N_{k}\left(x_{0}\right)}{\sum }K\left(x_{0},x_{i}\right)}. \end{array}$$

In Equation (12), *K*(*x*_0_, *x*_*i*_) is the kernel function evaluating the closeness of points *x*_0_ and *x*_*i*_, and *y*_*i*_ are the response values of the neighbors. Note that all *x*_*i*_ are needed to be rescaled to be in [0, 1]. This scaling not only helps remove unit dominance but also easily helps determine the bandwidth value of the kernel functions.

4) *Determining Optimal k and Bandwidth*

The optimal *k* and bandwidth parameters are those that minimize the average prediction error estimated through a 5-fold cross-validation. Let's denote the set of candidate *k* values as {*k*_1_, *k*_2_, ..., *k*_*r*_} and the set of candidate bandwidths as {*h*_1_, *h*_2_, ..., *h*_*s*_}. The objective is to find the optimal *k*_*opt*_ and *h*_*opt*_ that yield the lowest estimated prediction error:


(13) $$\begin{array}{l} \left(k_{opt},h_{opt}\right)=\arg\underset{k_{i},h_{j}}{\;min}\;CV\left(k_{i},h_{j}\right). \end{array}$$

In Equation (13), *CV*(*k*_*i*_, *h*_*j*_) represents the cross-validation error estimated over multiple random splits of the dataset into training and validation sets. Because all variables are scaled between 0 and 1, the distance between any two points will also fall within a bounded interval. This boundedness allows for the selection of h based on the maximum distance within the *k*-nearest neighbors for each query point, specifically for *k* = 2, 3, 5, 7. This method ensures that *h* is sufficiently large to encompass all neighbors in the calculation, thus being responsive to the local structure of the data and accommodating areas of varying density. The optimal (*k*_*opt*_, *h*_*opt*_) is found from the grid {*k*_1_, *k*_2_, ..., *k*_*r*_} × {*h*_1_, *h*_2_, ..., *h*_*s*_}.

5) *Ensemble Prediction*

The ensemble's predictive power is harnessed by aggregating the individual KNN models' outputs. If each model provides a prediction ŷ_*b*_ for *x*_0_. The final prediction is an aggregate statistic (e.g., mean) of these predictions, as shown in Equation (14):


(14) $$\begin{array}{l} {ŷ}_{final}=\frac{1}{B}\sum_{b=1}^{B}{ŷ}_{b}. \end{array}$$

The pseudocode below (Algorithm 1) concretizes the sequence of steps—from bootstrap sampling to the ensemble prediction—that collectively forge our proposed method.

**Algorithm 1:**
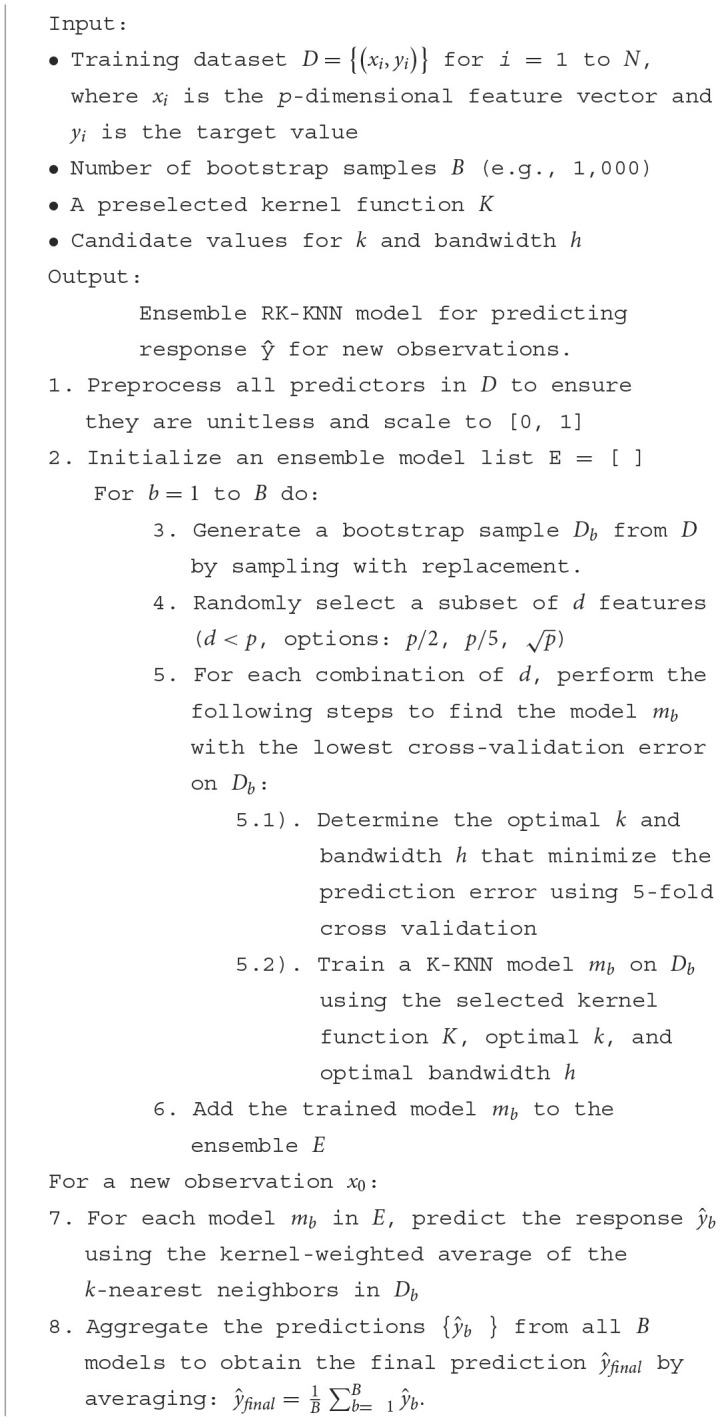
RK-KNN model for predicting responses.

## 4 Evaluation datasets and results

This section is dedicated to presenting the datasets used for benchmarking and the outcomes of the empirical evaluation conducted to assess the effectiveness of the RK-KNN regression approach. Additionally, state-of-the-art methods, including RF, ANN, and SVR, will be compared with the RK-KNN models.

### 4.1 Datasets for benchmarking

For assessing the new approach alongside existing leading techniques, we utilize 15 distinct datasets. These collections of data are acquired from multiple publicly accessible platforms. An overview of each dataset is presented in Table 1, detailing the number of observations (*n*), the number of predictor variables (*p*), and the meaning of the response variable.

**Table 1:** Datasets employed for model evaluation in RK-KNN regression with different kernels.

	**Dataset**	***n***	***p***	**Responses**
D1	Student performance (Cortez, 2014)	649	30	Math scores
D2	Student performance (Cortez, 2014)	649	30	Portuguese scores
D3	Wisconsin Prognostic Breast Cancer (Wolberg et al., 1995)	198	34	Recurrence time
D4	Properties of poly-aromatic hydrocar-bons (PAH) (Todeschini et al., 1995)	80	113	Effectiveness of the PDGFR inhibitors
D5	Platelet derived growth factor receptor (PDGFR) (Guha and Jurs, 2004)	79	304	Biological activity reported as IC50values
D6	Triazines (Hirst et al., 1994)	186	60	Inhibitory activity of triazine compounds
D7	Phenethyl (Kubinyi, 1993)	22	629	Phenethyl derivatives
D8	Topo (Feng et al., 2003)	8,885	267	Toxic effects from the compound structure
D9	Tecator (Borggaard, 1992; Thodberg, 1996)	240	125	Fat content of a meat sample
D10	Fric4 (Friedman, 1999)	1,000	100	Artificially generated responses from the model.
D11	HappinessRank (Helliwell et al., 2017)	235	9	Happiness scores from The World Happiness Report
D12	AutoHorse (OpenML, 2024)	201	69	Price
D13	Residential Building (Rafiei, 2018)	372	108	Actual sales prices
D14	Communities and Crime (Redmond, 2009)	1,994	100	Total number of violent crimes per 100K population
D15	Pumadyn (Alcalá-Fdez et al., 2011)	8,192	32	Angular acceleration of one of the robot arms

### 4.2 Performance evaluation

The performance of the RK-KNN method, when compared to the standard KNN and R-KNN, across datasets D1 to D15, is summarized in Table 2. It reveals the effectiveness of the RK-KNN method in enhancing predictive accuracy. The RK-KNN method, particularly when employing specific kernels, consistently outperforms the standard KNN in terms of root mean square serror (RMSE), mean absolute error (MAE), and R-squared (R^2^) values.

**Table 2:** Performance evaluation of KNN, R-KNN, and RK-KNN with various kernel functions across datasets (bold values represent the best performance).

	**Method**	**RMSE**	**MAE**	**R^2^**
D1	KNN	4.1878	3.1845	0.2055
	R-KNN	3.7979	2.7844	0.6291
	**RK-KNN with kernel:**			
	Gaussian	3.7976	2.7844	0.6287
	Epanechnikov	3.7975	2.7846	0.6288
	Uniform	3.7979	2.7844	**0.6304**
	Triangular	**3.7949**	**2.7828**	0.6284
	Quartic	3.7971	2.7848	0.6275
	Tricube	3.7982	2.7856	0.6273
D2	KNN	2.8320	2.0736	0.2579
	R-KNN	2.5161	1.7633	0.6392
	**RK-KNN with kernel:**			
	Gaussian	2.4875	1.7457	0.6434
	Epanechnikov	2.4873	1.7445	0.6432
	Uniform	2.4878	1.7450	0.6434
	Triangular	**2.4852**	**1.7429**	**0.6436**
	Quartic	2.4867	1.7441	0.6431
	Tricube	2.4874	1.7447	0.6429
D3	KNN	**31.9275**	**27.5843**	**0.1452**
	R-KNN	32.0872	27.8724	0.1352
	**RK-KNN with kernel:**			
	Gaussian	32.0903	27.8737	0.1348
	Epanechnikov	32.0473	27.8755	0.1341
	Uniform	32.0335	27.8629	0.1352
	Triangular	32.0549	27.8799	0.1333
	Quartic	32.0614	27.8793	0.1330
	Tricube	32.0629	27.8772	0.1331
D4	KNN	0.103200	0.088300	0.7049
	R-KNN	0.100200	0.081800	0.7194
	**RK-KNN with kernel:**			
	Gaussian	0.100027	0.082446	0.7202
	Epanechnikov	0.099257	0.081848	0.7247
	Uniform	0.099807	0.082148	0.7224
	Triangular	0.098940	0.081626	0.7260
	Quartic	**0.098797**	**0.081580**	**0.7265**
	Tricube	0.098812	0.081613	**0.7265**
D5	KNN	0.1811	0.1302	0.4476
	R-KNN	0.1749	0.1242	0.4726
	**RK-KNN with kernel:**			
	Gaussian	0.1745	0.1239	0.4752
	Epanechnikov	0.1733	0.1228	0.4818
	Uniform	0.1749	0.1242	0.4726
	Triangular	0.1729	0.1223	0.4844
	Quartic	0.1723	0.1217	0.4879
	Tricube	**0.1722**	**0.1216**	**0.4886**
D6	KNN	0.1391	0.1028	0.2026
	R-KNN	0.1321	0.0970	0.2691
	**RK-KNN with kernel:**			
	Gaussian	0.1320	0.0969	0.2696
	Epanechnikov	0.1320	0.0969	0.2702
	Uniform	0.1321	0.0970	0.2691
	Triangular	**0.1318**	**0.0967**	**0.2720**
	Quartic	0.1319	0.0968	0.2713
	Tricube	0.1319	0.0969	0.2705
D7	KNN	0.1461	0.1233	0.8585
	R-KNN	0.1454	0.1225	0.8563
	**RK-KNN with kernel:**			
	Gaussian	0.1444	0.1223	0.8566
	Epanechnikov	0.1406	0.1188	0.8646
	Uniform	0.1429	0.1213	0.8635
	Triangular	0.1400	0.1180	0.8651
	Quartic	0.1389	0.1171	0.8652
	Tricube	**0.1387**	**0.1169**	**0.8655**
D8	KNN	0.0290436	0.0203419	0.0320561
	R-KNN	0.0280759	0.0195862	0.0519505
	**RK-KNN with kernel:**			
	Gaussian	0.0280758	0.0195861	0.0519602
	Epanechnikov	0.0280756	0.0195860	0.0520021
	Uniform	0.0280759	0.0195862	0.0519789
	Triangular	**0.0280745**	**0.0195852**	**0.0520842**
	Quartic	0.0280753	0.0195857	0.0520282
	Tricube	0.0280756	0.0195860	0.0520045
D9	KNN	4.9607	3.6138	0.9043
	R-KNN	4.8846	3.5413	0.9189
	**RK-KNN with kernel:**			
	Gaussian	4.8699	3.5324	0.9192
	Epanechnikov	4.8446	3.5176	0.9197
	Uniform	4.8839	3.5412	0.9189
	Triangular	4.8103	**3.4896**	**0.9205**
	Quartic	**4.8101**	3.4962	0.9204
	Tricube	4.8204	3.5057	0.9202
D10	KNN	0.9900	0.7839	0.0421
	R-KNN	0.9532	0.7586	0.1469
	**RK-KNN with kernel:**			
	Gaussian	0.9532	0.7586	0.1464
	Epanechnikov	0.9525	0.7580	0.1440
	Uniform	0.9526	0.7581	0.1468
	Triangular	0.9526	0.7579	0.1436
	Quartic	0.9525	0.7579	0.1407
	Tricube	**0.9524**	**0.7578**	**0.1382**
D11	KNN	0.3953	0.3048	0.8877
	R-KNN	0.3802	0.2838	0.8999
	**RK-KNN with kernel:**			
	Gaussian	0.3799	0.2837	0.9000
	Epanechnikov	0.3760	0.2818	0.9023
	Uniform	0.3767	0.2821	0.9020
	Triangular	0.3754	**0.2814**	0.9025
	Quartic	**0.3753**	0.2815	**0.9026**
	Tricube	0.3756	0.2818	0.9024
D12	K-NN	3,051.2	2,105.2	0.8507
	R-KNN	2,983.6	1,960.6	0.8810
	**RK-KNN with kernel:**			
	Gaussian	2,961.1	1,949.2	0.8824
	Epanechnikov	2,919.6	1,928.6	0.8848
	Uniform	2,983.6	1,960.6	0.8810
	Triangular	2,879.4	1,907.3	0.8871
	Quartic	**2,871.2**	**1,904.3**	**0.8872**
	Tricube	2,877.5	1,907.5	0.8869
D13	KNN	730.86	486.82	0.5993
	R-KNN	710.57	475.59	0.6254
	**RK-KNN with kernel:**			
	Gaussian	710.20	475.36	0.6255
	Epanechnikov	710.71	475.52	0.6247
	Uniform	710.57	475.55	0.6254
	Triangular	**707.66**	**473.38**	**0.6272**
	Quartic	710.54	475.21	0.6243
	Tricube	712.53	476.36	0.6224
D14	KNN	0.143249	0.0968667	0.62008
	R-KNN	0.141207	0.0969202	0.64373
	**RK-KNN with kernel:**			
	Gaussian	0.141195	0.0969090	0.64377
	Epanechnikov	0.141183	0.0968933	0.64380
	Uniform	0.141207	0.0969201	0.64373
	Triangular	0.141162	0.0968674	0.64385
	Quartic	**0.141160**	**0.0968671**	**0.64387**
	Tricube	0.141171	0.0968790	0.64385
D15	KNN	0.027170	0.021513	0.189703
	R-KNN	0.026824	0.021045	0.343268
	**RK-KNN with kernel:**			
	Gaussian	0.026820	0.021042	0.343363
	Epanechnikov	0.026806	0.021032	0.343543
	Uniform	0.026822	0.021044	0.343268
	Triangular	0.026802	0.021029	0.343651
	Quartic	0.026790	0.021020	0.343954
	Tricube	**0.026784**	**0.021016**	**0.344055**

Overall, the triangular kernel emerges as the most effective, closely followed by the Tricube kernel. This observation is supported by instances across multiple datasets; for example, in dataset D1, the triangular kernel achieves an RMSE of 3.7949, an MAE of 2.7828, and an R^2^ of 0.6304, surpassing the performance of classical KNN. Similarly, in dataset D6, the triangular kernel demonstrates superior results with an RMSE of 0.1318, an MAE of 0.0967, and an R^2^ of 0.272.

The Gaussian and Epanechnikov kernels tend to not give the lowest RMSE or MAE but still perform notably well compared to the traditional KNN and R-KNN. The uniform kernel sometimes shows superiority compared to the other kernel functions.

**Figure 3:**
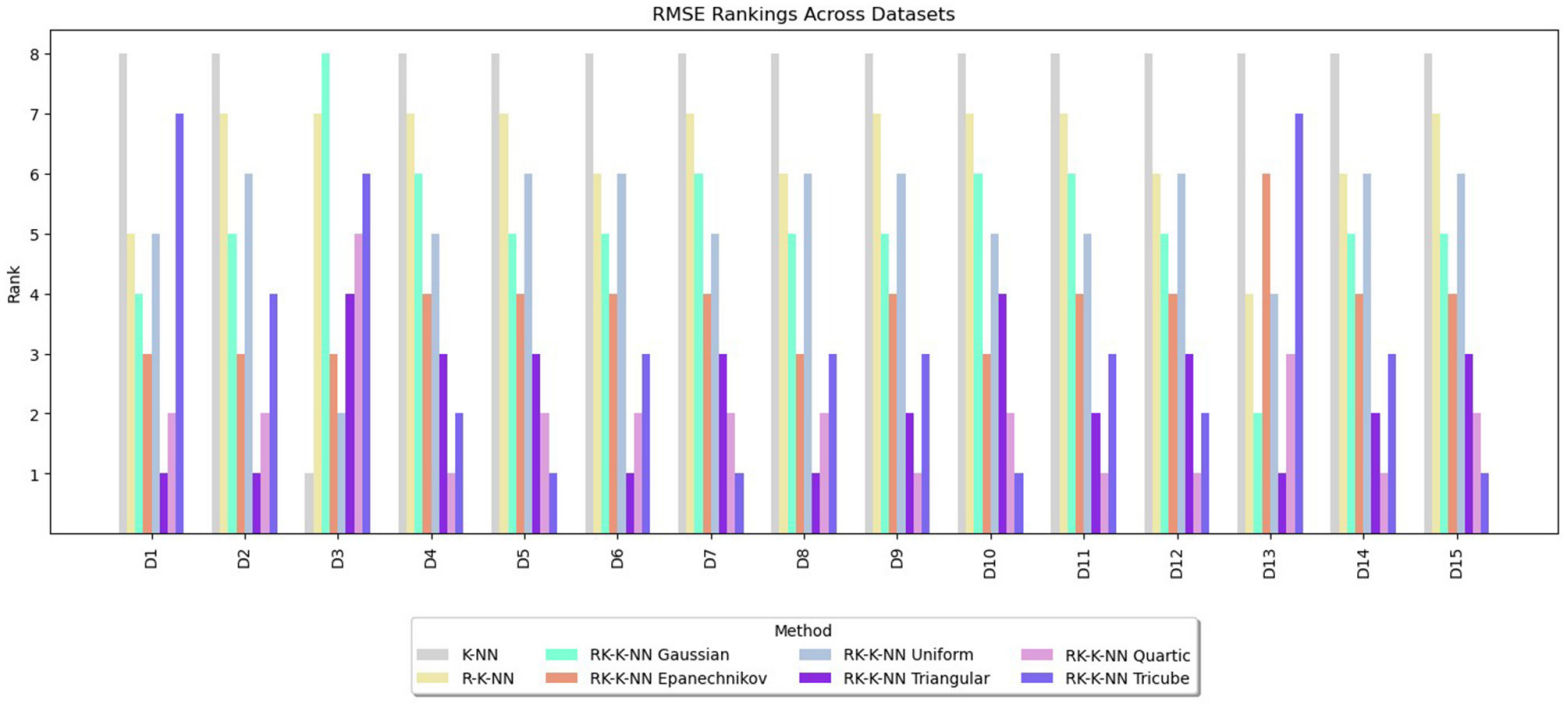
Comparative performance of RK-KNN with different kernel functions, R-KNN, and KNN regressions on multiple datasets using RMSE rankings.

**Figure 4:**
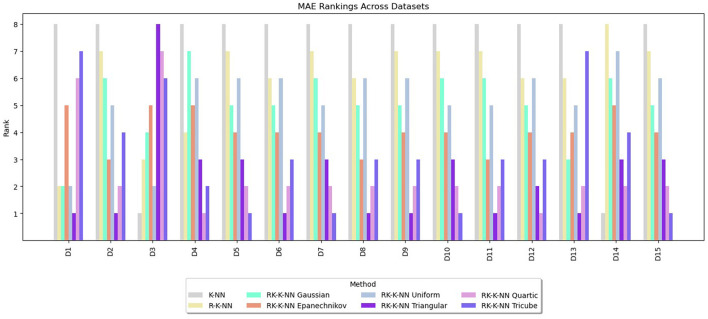
Comparative performance of RK-KNN with different kernel functions, R-KNN, and KNN regressions on multiple datasets using MAE rankings.

**Figure 5:**
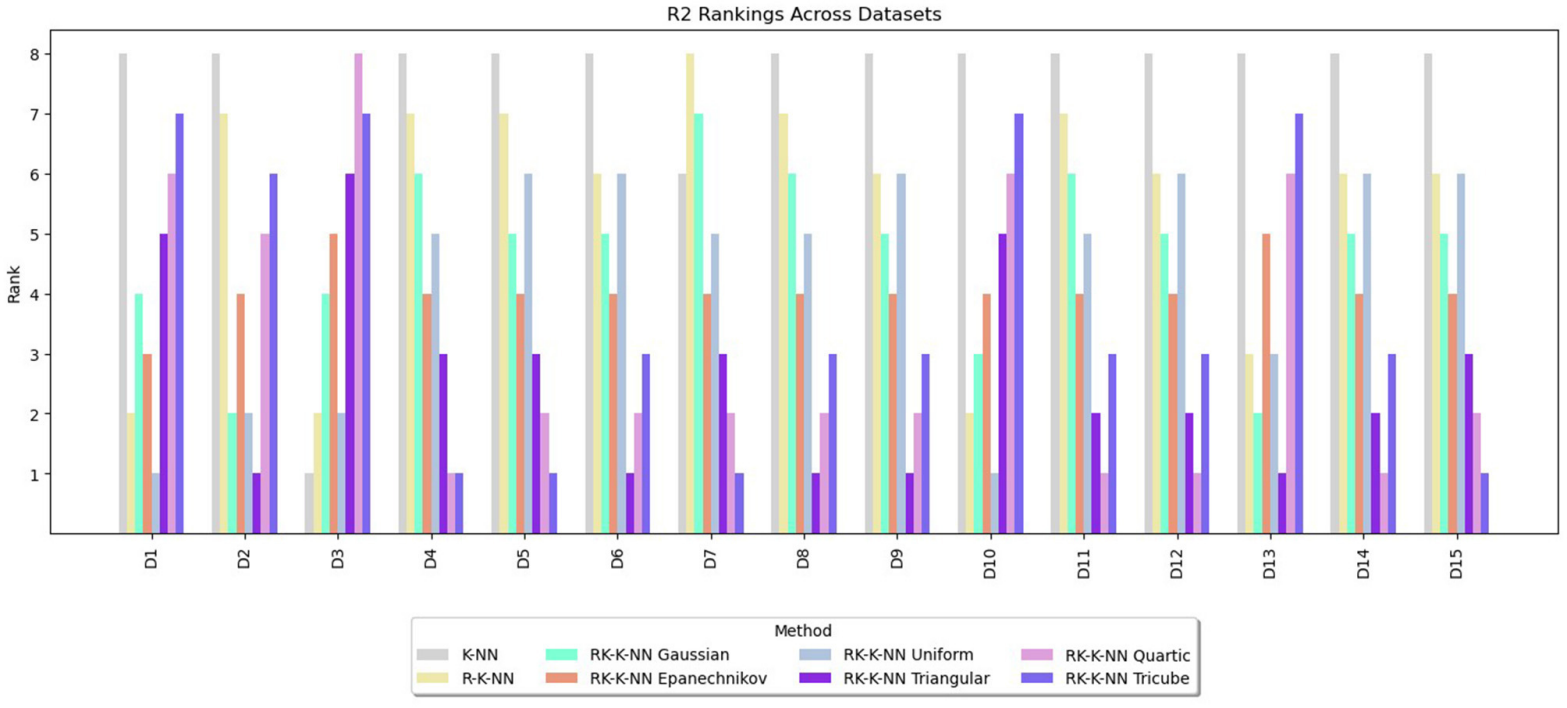
Comparative performance of RK-KNN with different kernel functions, R-KNN, and KNN regressions on multiple datasets using R^2^ rankings.

Rankings are assigned to the methods from one to eight based on the values of RMSE, MAE, and R^2^. The lowest RMSE or MAE values receive a rank of one, indicating the best performance, while for R^2^, the highest value is awarded a rank of one. The rankings for RMSE, MAE, and R^2^ are summarized in Figures 3–5, respectively. These graphs demonstrate that the RK-KNN regression models generally achieve lower ranks, indicating better performance compared to the R-KNN and traditional KNN regression models. Specifically, for RMSE, the average ranks for RK-KNN with quartic, triangular, tricube, Epanechnikov, Gaussian, and uniform kernels are 1.93, 2.27, 3.13, 3.80, 5.20, and 5.27, respectively. In contrast, the R-KNN and traditional KNN models have average ranks of 6.40 and 7.53, respectively.

For MAEs, RK-KNN models with triangular and quartic kernels exhibit nearly identical rankings, with average ranks of 2.33 and 2.67, respectively. The rankings for other methods are consistent with those observed for RMSE. The sequence features RK-KNN with tricube, Epanechnikov, Gaussian, and uniform kernels, followed by R-KNN and KNN, with respective average ranks of 3.27, 4.07, 5.07, 5.20, 6.00, and 7.07.

For R^2^, the RK-KNN model using the triangular kernel shows the best performance, achieving the lowest average rank of 2.60. It is followed by the RK-KNN models with quartic, tricube, Epanechnikov, uniform, and Gaussian kernels. R-KNN and KNN lag behind, with average ranks for R^2^ being 3.13, 3.73, 4.07, 4.33, 4.67, 5.47, and 7.40, respectively.

### 4.3 Comparisons with state-of-the-art methods

The KNN models exhibiting the lowest RMSEs were benchmarked against RF, ANN, and SVR across fifteen diverse datasets, as detailed in Table 3. KNN-typed learners showed superior performance in datasets D3, D4, D5, D7, and D8, representing a third of the datasets. However, they were notably outperformed by RF in nine datasets (D1, D2, D6, D9, D10, D12, D13, D14, and D15) and by ANN and SVR in the remaining datasets. Although RK-KNN regression did not achieve the lowest RMSE in all datasets, it remains a competitive option, particularly against SVR and ANN. This is especially evident in datasets D5 and D8, which contain a high number of features, where RK-KNN was preferred over the other models.

**Table 3:** Performance evaluation of best KNN-typed learner, RF, ANN, and SVR (bold values represent the best performance).

	**Method**	**RMSE**	**MAE**	**R^2^**
D1	Random Kernel KNN	3.7949	2.7828	0.6284
	**Random Forest**	**1.9710**	**1.1991**	**0.8105**
	Artificial Neural Network	2.8195	2.1649	0.6123
	Support Vector Regression	2.9470	1.9306	0.5864
D2	Random Kernel KNN	2.4852	1.7429	0.6436
	**Random Forest**	**1.2806**	**0.8084**	**0.8384**
	Artificial Neural Network	1.7284	1.2457	0.7065
	Support Vector Regression	1.7219	1.0574	0.7138
D3	**KNN**	**31.9275**	**27.5843**	**0.1452**
	Random Forest	34.0136	28.8079	0.0326
	Artificial Neural Network	46.5660	37.2240	−0.8355
	Support Vector Regression	34.8618	28.7294	−0.0121
D4	**Random Kernel KNN**	**0.0987**	**0.0815**	**0.7265**
	Random Forest	0.1048	0.0854	0.6867
	Artificial Neural Network	0.2081	0.1484	−0.5068
	Support Vector Regression	0.1401	0.1057	0.6459
D5	**Random Kernel KNN**	**0.1722**	**0.1216**	**0.4886**
	Random Forest	0.1826	0.1297	0.2731
	Artificial Neural Network	4.9656	1.5578	−1,988.9871
	Support Vector Regression	0.1945	0.4237	0.2521
D6	Random Kernel KNN	0.1318	0.0967	0.2720
	**Random Forest**	**0.1243**	**0.0912**	**0.3758**
	Artificial Neural Network	0.2709	0.1862	−1.9643
	Support Vector Regression	0.1398	0.1003	0.2095
D7	**Random Kernel KNN**	**0.1387**	**0.1169**	**0.8655**
	Random Forest	0.1486	0.1208	0.3686
	Artificial Neural Network	3.3553	2.2652	−2,239.9117
	Support Vector Regression	0.2118	0.1659	−0.2131
D8	**Random Kernel KNN**	**0.0280**	**0.0195**	**0.0520**
	Random Forest	0.0297	0.0201	0.0526
	Artificial Neural Network	0.1546	0.0746	−29.6140
	Support Vector Regression	0.0376	0.0297	−0.5931
D9	Random Kernel KNN	4.8103	3.4896	0.9205
	**Random Forest**	**1.5273**	**1.0882**	**0.9883**
	Artificial Neural Network	3.8876	2.6226	0.9292
	Support Vector Regression	11.1648	7.9844	0.4088
D10	Random Kernel KNN	0.9524	0.7578	0.1382
	**Random Forest**	**0.3749**	**0.2891**	**0.8640**
	Artificial Neural Network	1.0516	0.8608	−0.0693
	Support Vector Regression	0.9534	0.7723	0.1211
D11	Random Kernel KNN	0.3753	0.2815	0.9026
	Random Forest	0.3839	0.2803	0.8846
	Artificial Neural Network	0.5566	0.4287	0.7311
	**Support Vector Regression**	**0.3022**	**0.1816**	**0.9238**
D12	RK-KNN with Quartic	2,871.2	1,904.3	0.8872
	**Random Forest**	**2,145.3**	**1,470.5**	**0.8921**
	Artificial Neural Network	12,614.1	11,164.4	−2.2913
	Support Vector Regression	7,493.0	4,895.2	−0.1041
D13	RK-KNN with Triangular	707.66	473.38	0.6272
	**Random Forest**	**246.12**	**129.67**	**0.9564**
	Artificial Neural Network	198.85	116.84	0.9712
	Support Vector Regression	1,252.86	805.01	−0.0971
D14	Random Kernel KNN	0.141160	0.0968671	0.64387
	**Random Forest**	**0.136293**	**0.0939809**	**0.64433**
	Artificial Neural Network	0.196391	0.1486414	0.26220
	Support Vector Regression	0.142445	0.1037052	0.61202
D15	Random Kernel KNN	0.026784	0.021016	0.34405
	**Random Forest**	**0.007750**	**0.006169**	**0.93407**
	Artificial Neural Network	0.066096	0.052390	−3.87338
	Support Vector Regression	0.030218	0.023477	−0.00120

## 5 Conclusion and discussion

This study validates the efficacy of integrating kernel functions with a random process, which includes both bootstrapping and feature selection, across 15 datasets. Our comprehensive evaluation, based on criteria such as RMSE, MAE, and R^2^, underscores the superiority of the RK-KNN approach, especially when employing quartic, triangular, and tricube kernel functions. These kernels have consistently demonstrated performance enhancements across various case studies.

**Figure 6:**
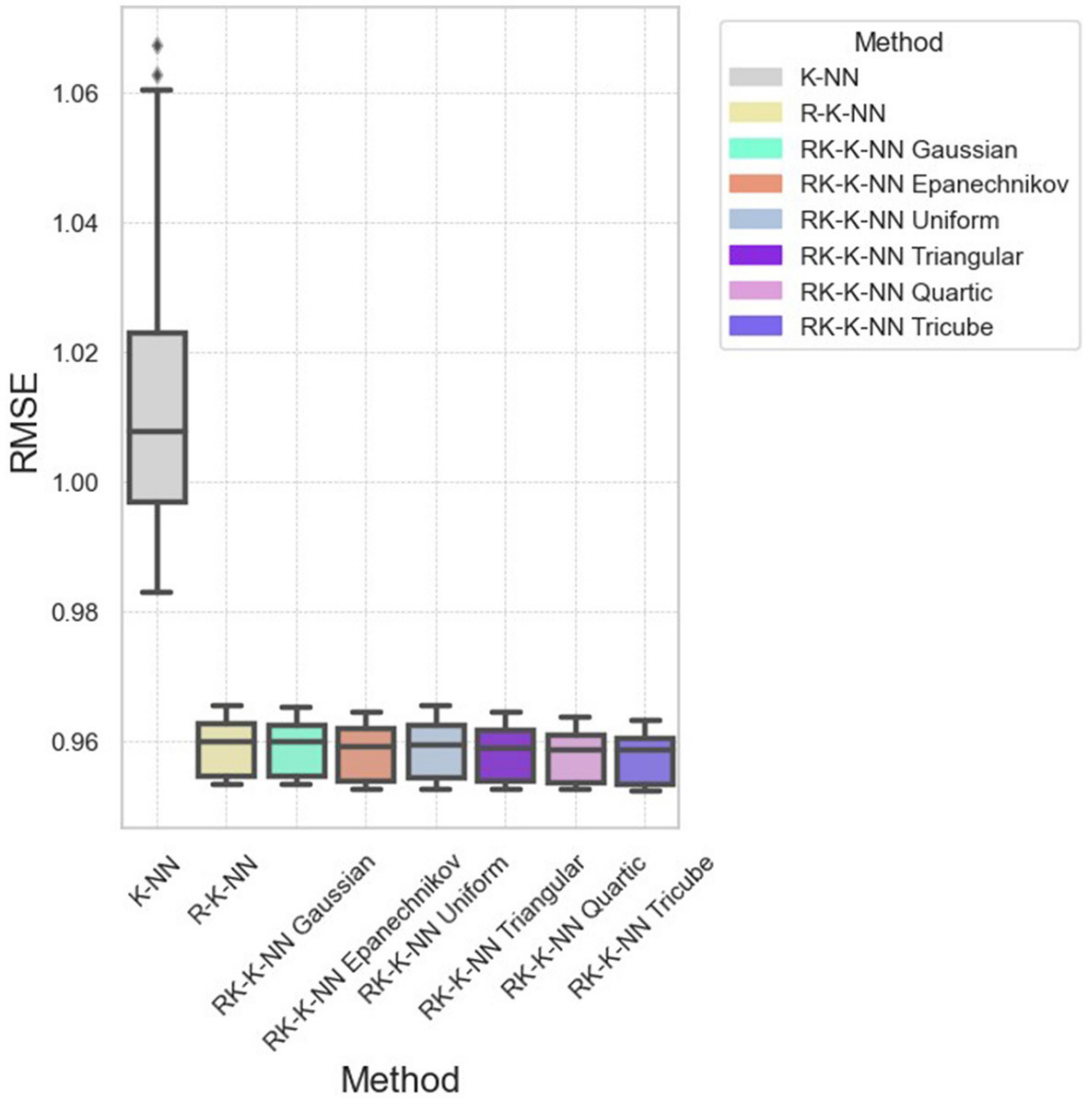
The RMSE distribution across all eight methods for dataset D10.

**Figure 7:**
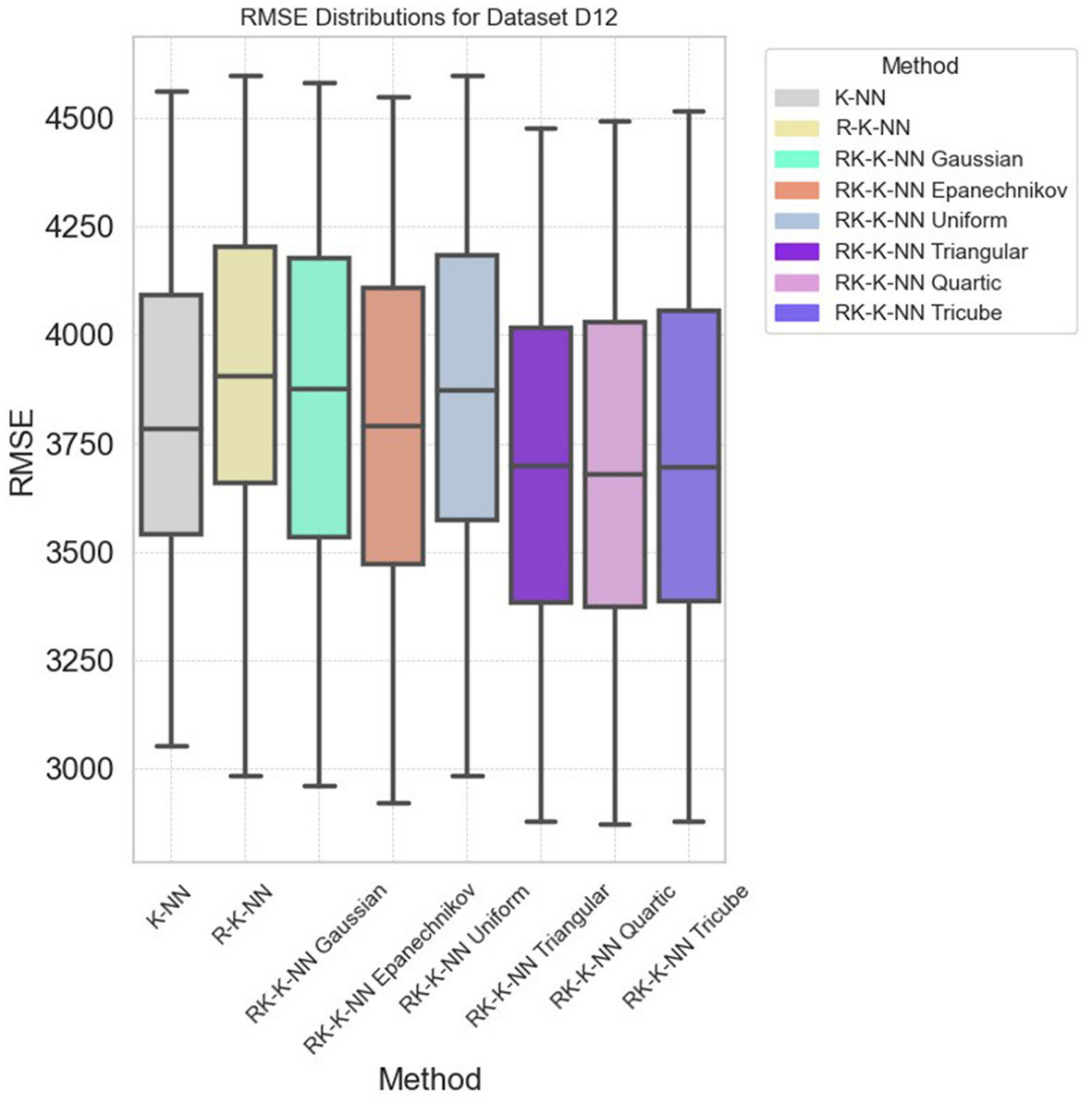
The RMSE distribution across all eight methods for dataset D12.

Specifically, in dataset D10, RK-KNN regression markedly improves prediction accuracy. The RMSE distributions depicted in Figure 6 reveal that standard KNN exhibits higher RMSE compared to other methods, with all parameter configurations for RK-KNN outperforming standard KNN. However, achieving optimal performance across datasets may require a comprehensive search to identify the best parameters for the selected kernel functions. As shown in Figure 7, while the lowest RMSE values for RK-KNN across all kernel functions are superior to those of KNN, the medians of RMSEs for some kernels, like the Gaussian kernel, exceed the median RMSE of KNN. This variability indicates a critical need for tuning the optimal bandwidth and *k*-value to consistently achieve the lowest RMSE.

Moreover, the computational cost and scalability of the RK-KNN algorithm's cross-validation process are effectively managed through vectorized distance computations, which enhance calculation speed and reduce runtime. Standardization of features further contributes to this efficiency by simplifying the distance metric computation. A well-controlled grid search for parameter tuning, along with the ability to independently execute bootstrapping and feature selection steps, ensures computational tractability. Practical applications across multiple datasets have demonstrated that the cross-validation step, a critical aspect of the RK-KNN algorithm, is not prohibitively time-consuming. Therefore, the RK-KNN method is computationally efficient and well-suited for the analysis of large-scale data environments.

## Data availability statement

The original contributions presented in the study are included in the article/supplementary material, further inquiries can be directed to the corresponding author.

## Author contributions

PS: Conceptualization, Funding acquisition, Investigation, Methodology, Project administration, Writing – original draft, Writing – review & editing. KS: Data curation, Formal analysis, Resources, Software, Visualization, Writing – review & editing.
